# Increase of vitamin D assays prescriptions and associated factors: a population-based cohort study

**DOI:** 10.1038/s41598-017-10263-8

**Published:** 2017-09-04

**Authors:** Pascal Caillet, Anne Goyer-Joos, Marie Viprey, Anne-Marie Schott

**Affiliations:** 1 Hospices Civils de Lyon, Pôle IMER, Lyon, F-69003 France; 2 Lyon University, INSERM EA HESPER 7425, F-69003 Lyon, France

## Abstract

A worldwide increase in the frequency of testing for serum 25-hydroxyvitamin D (25OHD) levels has been observed over the last years. Our aim was to measure the evolution in the number of vitamin D assays performed in France from 2008 to 2013 and to investigate some of the drivers that may explain this increase. Patients within the representative 1/97th sample of the French health insurance system reimbursement database (*EGBS* database) who had at least one 25OHD or 1-25(OH)_2_D assay between 2008 and 2013 were included. Trends over time in number of vitamin D assays were analysed globally and per year in a multivariable Poisson regression model with GEE. Among the 639,163 patients of the *EGBS* database, 118,509 (18.5%) had at least one vitamin D assay over the 6-year study period. Among the individuals tested, 52.1% had only one test. The number of vitamin D assays (25OHD or 1-25(OH)_2_D) increased 7.5-fold from 9,620 in 2008 to 81,641 in 2013. This study confirms the rapid and dramatic increase in vitamin D assays prescriptions and shows that this is mostly due to a global increase of the proportion of patients tested rather than an increase in repetition of tests in some individual patients.

## Introduction

Vitamin D deficiency is known to be involved in osteomalacia in adults and rickets in children. A low serum concentration of 25-hydroxyvitamin D (25OHD) is also considered as a risk factor for osteoporosis^1^. Historically, vitamin D assays were typically prescribed in the context of bone metabolism assessment^2^. Many observational studies have described the potential non-skeletal effects of vitamin D, including an important list of chronic disorders (e.g., cancer, cardiovascular disease, diabetes, and autoimmune disorders). Nevertheless, the evidence that deficiency is associated with increased risk of these diseases is still debated since experimental studies show conflicting results^3^. Against the background of this growing interest in the pleiotropic effects of vitamin D, a worldwide massive increase in demand for measurement of the serum 25OHD level from the public and physicians was observed over recent years in several countries^4–6^. In France, the number of assays reimbursed increased from 1.5 million dosages in 2009 to 4.5 million in 2011 leading to a dramatic increase in the global costs reimbursed from €27 million in 2009 to €65 million in 2011^7^. Although the French Health Insurance Fund progressively decreased the reimbursement rate of 25OHD testing which led to a decrease of unit pricing the total amount spent was almost multiplied by 3 within 2 years. In this context, our main objective was to describe the patterns of this observed increase in vitamin D assays, its possible change over time, and the associated factors. In particular we wanted to investigate whether this increase was linear or exponential over time, whether this was specific of some subpopulations of patients or some medical specialties, and whether this was linked to an increase of the proportion of patients prescribed one assay or an increase in assays repetitions in some individuals.

## Methods

### Data source

We used the database of the “*Echantillon Généraliste de Bénéficiaires simplifié” (EGBS)*, a permanent representative sample of the general population of subjects affiliated with the French National Health Insurance Fund^8^. The *EGBS* includes healthcare consumptions of beneficiaries covered by the main scheme of the Health Insurance Fund for Salaried Workers (*CNAMTS*). Since 2011, the *EGBS* includes also beneficiaries from the National Health Insurance Fund for Agricultural Workers and Farmers (*MSA*) and the National Health Insurance Fund for the Self-employed (*RSI*), increasing the representativeness to 85.5% of the French population. This database currently includes more than 600,000 patients and contains all their reimbursements data for hospitalizations, drugs and tests ordered by physicians. The French Healthcare Insurance Fund has already been described elsewhere^9^.

### Study sample

An cohort was constituted from EGBS database and included all individuals registered in the *EGBS* from January 1^st^, 2008 to December 31^th^, 2013 who had at least one 25OHD or 1-25(OH)_2_D assay during this period. Exclusion criteria were death or exclusion from the *EGBS* during the study period. Realisation of vitamin D assays was identified by a reimbursement for a procedure with the code 1139 (25OHD) or 1820 (1-25(OH)_2_D) according to the French registry of health procedures (“*Table Nationale de codage de Biologie*”).

We anonymously extracted the following demographic data from the database: sex, date of birth, department of residence, prescriber of vitamin D assay (specialty, type of practice), associated reimbursed biological tests, chronic disease status *(“Affection de longue durée” (ALD)* status) and universal health care insurance status (“*Couverture maladie universelle*” (*CMU*) status). The *CMU* status reflects the socioeconomic group of the patient, as it is attributed to patients with low income. The *ALD* status identifies patients with a major chronic disease and is coded according to the International Classification of Disease, 10^th^ version (ICD-10) classification system, as declared by their general practitioner (GP) and approved by a physician employed by the National Healthcare Insurance Fund^10^. The Charlson index was computed by the use of ICD-10 codes recorded during the last hospitalization when available^11^. An osteoporosis medical management was evaluated by the presence of a reimbursement for osteoporosis pharmacotherapy (OP) during the year. The list of drugs used is described in supplementary material.

### Statistical analysis

Results are presented as frequencies and percentage of subjects for qualitative variables and as mean values with standard deviation for quantitative variables. Comparisons of means between the sample and the *EGBS* population were performed by use of Welch-test for continuous variable (assuming inequality of variance) and chi-square test for categorical variables. The annual global number of prescriptions was aggregated by calendar year from 2008 to 2013 within the sample. Vitamin D assays prescription evolution over time in the sample was described according to the type of physician who issued the prescription, patient’s age at inclusion, sex, context of care (*ALD* status), Charlson Index, reimbursment for OP, and low income status (*CMU* status). The statistical units considered were vitamin D assays. Formal tests of changes in distribution over time in patients’age, type of assay, and prescriber specialty were performed by use of Chi-square tests.

Additionally, patients who had at least one 25OHD or 1-25(OH)_2_D assay during the study period were followed longitudinally to assess the repetition of measures over time in single individuals and document hypotheses regarding drivers of this repetition. The statistical units considered were patients-year. A multivariable regression analysis was performed. The model was a Poisson regression model with generalized estimating equation (GEE), an approach developed by Liang and Zeger^12^, which accounts for the correlation between measurement counts in a single individual followed longitudinally. The cluster was specified to be at the patient level and an unstructured working correlation matrix was used to account for the correlation pattern between vitamin D assays reimbursements within the same patient over time. Overdispersion was checked. Covariates included age at inclusion, sex, presence of *ALD* status during the year, Charlson Index, presence of OP during the year, presence of *CMU* status during the year and time (year) as independent variables. The annual number of vitamin D assays was the dependent variable. Incident rate ratios (IRR) adjusted on independent variables were computed with their 95% confidence intervals. Data were controlled, validated and analysed using SAS® Enterprise Guide Software V.4.3 (SAS Institute Inc., Cary, NC, USA). Analyses in EGBS have been approved by the French “Commission Nationale de l’Informatique et des Libertés” (CNIL).

## Results

### Study sample

Among the 639,163 patients included in the *EGBS* database over the study period (January 2008-December 2013), 118,509 (18.5%) underwent at least one vitamin D assay and were included in the cohort. Characteristics of these patients are shown in Table 1. Compared to the total *EGBS* database (i.e the source population), patients who had at least one vitamin D assay over the study period were older (mean age of 58 years vs. 36 years in the *EGBS*) and more frequently women (73.9% vs. 50.6% in the *EGBS* database). Regarding their health status, 34.9% of the study patients had a chronic disease (*ALD* status) versus 17.1% in the *EGBS* database. Inversely, fewer patients had a *CMU* status in the study sample (5.0%) compared to the *EGBS* database (13.8%). Half of the study population had only one assay over the 6-year period (52.1%, 61,763 patients). The other half had more than one assay, roughly one quarter (22.2%) had two assays and one quarter (25.6%) had three assays or more over the 6-year study period.

**Table 1:** Characteristics of the study population and the source population (*EGBS)*.

	Patients with at least one 25OHD assay	*Source population (EGBS)*	p-value
Patients, n	118,509	639,163	
Female gender, n (%)	87,673 (73.9)	323,535 (50.6)	<0.001
Age at inclusion (mean, SD)	57.9 (17.8)	35.7 (23.9)	<0.001
Age at inclusion, n (%)			
0–18	0	173,072 (27.1)	<0.001
18– < 30	8,394 (70.8)	100,421 (15.7)	
30– < 40	12,036 (10.1)	87,593 (13.7)	
40– < 50	17,174 (14.5)	86,307 (13.5)	
50– < 60	23,858 (20.1)	75,546 (11.8)	
60– < 70	23,760 (20.0)	53,184 (8.3)	
70– < 80	18,120 (15.3)	39,548 (6.2)	
80– < 90	12,754 (10.8)	20,477 (3.2)	
≥90 y	2,413 (0.2)	2,982 (0.4)	
Charlson Index, mean (SD)	0.49 (1.51)	0,21 (0.92)	<0.001
Chronic disease status (*ALD* status), n (%)	41,355 (34.9)	109,187 (17.1)	<0.001
Presence of OP, n (%)	13,729 (11.6)	19,506 (3.0)	<0.001
Low economic resources status (*CMU* status), n (%)	6,872 (7.6)	80,043 (12.5)	<0.001

Legend: This table presents the characteristics of the patients included in the study sample, i.e with at least one 25OHD assay during the study period, compared to the characteristics of the patients included in the whole EGBS database at the time of the extraction (May 2016).

### Characteristics of trends in assay of vitamin D

During the study period, the annual number of 25OHD assays increased approximately 8-fold (from 9,620 in 2008 to 80,297 in 2013) and the number of 1-25(OH)_2_D increased 3-fold (from 455 in 2008 to 1,344 in 2013). Concurrently, the annual number of patients receiving at least one assay increased in a similar scale (9-fold) from 5,420 in 2008 to 48,841 in 2014 (Table 2). Taking into account the global increase of the *EGBS* population over the 6-year period, the proportion of patients receiving at least one assay over the period increased from 1.4% in 2008 to 10.2% in 2013. The increase was mostly associated with prescriptions from GPs, which increased from approximately 5,200 to 53,700, and to a lesser extent with prescriptions from hospital specialists from 1,466 to 16,988. Most prescriptions were 25OHD assays, 95.3% in 2008 slightly increasing to 98.4% in 2013 (Table 2).

**Table 2:** Evolution of prescriptions characteristics between 2008 and 2013.

Year	2008	2009	2010	2011	2012	2013	p-value*
Total *EGBS*, n	503,758	507,251	526,108	594,370	602,199	609,205	
Study patients, n (%)	7,101 (1.4)	12,789 (2.5)	24,308 (4.6)	41,864 (7.0)	53,655 (8.9)	62,418 (10.2)	<0.001#
Number of assays prescribed, n	9,620	17,046	32,479	55,679	70,648	81,641	
Type of assay, n (%)							<0.001
25OHD	9,165 (95.3)	16,208 (95.1)	31,222 (96.1)	53,977 (96.9)	69,107 (97.8)	80,297 (98.4)	
1,25(OH)_2_D	455 (4.7)	838 (4.9)	1257 (3.9)	1702 (3.1)	1541 (2.2)	1344 (1.6)	
Type of prescriber, n (%)							<0.001
General practitioners	5,214 (54.2)	10,357 (60.8)	22,082 (68.0)	35,570 (63.9)	46,292 (65.5)	53,736 (65.8)	
Private specialists	2,922 (30.4)	4,387 (25.7)	6,676 (20.6)	8,571 (15.4)	9,810 (13.9)	10,691 (13.1)	
Public hospital practitioners	1,466 (15.2)	2,266 (13.3)	3,639 (11.2)	11,416 (20.5)	14,380 (20.4)	16,988 (20.8)	
Unknown	18 (0.2)	36 (0.2)	82 (0.3)	122 (0.2)	166 (0.2)	226 (0.3)	
Number of assays per patient, n (%)							<0.001
1	5,420 (76.3)	9,826 (76.8)	18,647 (76.7)	32,181 (76.9)	41,500 (77.3)	48,841 (78.2)	
2	1,234 (17.4)	2,193 (17.1)	4,095 (16.8)	7,147 (17.1)	9,126 (17.0)	10,076 (16.1)	
3	251 (3.5)	487 (3.8)	1,020 (4.2)	1,653 (3.9)	1,981 (3.7)	2,275 (3.6)	
4	106 (1.5)	171 (1.3)	341 (1.4)	565 (1.3)	694 (1.3)	775 (1.2)	
≥5	90 (1.3)	112 (0.9)	205 (0.8)	318 (0.8)	354 (0.7)	451 (0.7)	

*p-value for heterogeneity; # p-value for trend

Legend: This table presents the evolution of the fraction of EGBS database’s patients included receiving at least one 25OHD assay (study sample) according to year with a corresponding p-trend (rows 2 to 3), the context associated with the vitamin D assay prescription (rows 4 to 12) and the yearly number of assays prescribed by unique patient (rows 13 to 18).

**Figure 1:**
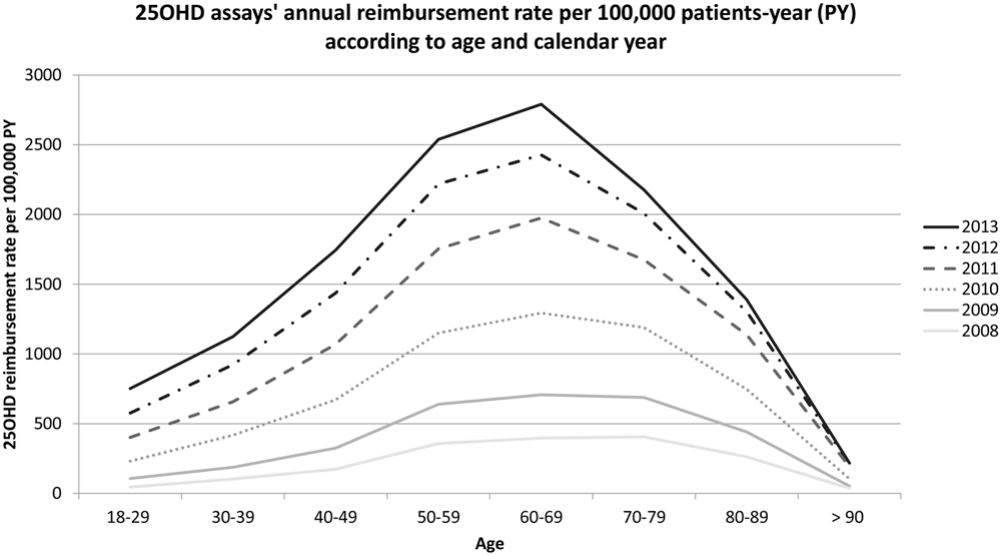
25OHD assays reimbursement annual rate per 100000 patients-year according to age and calendar year.

The number of assays prescribed per patient per year increased uniformly across the strata. The proportion of patients having 1, 2, 3, 4, or 5 or more assays within each year remained stable over the 6-year period. Every year, among patients receiving an assay, roughly 80% had only one test over the year, and less than 5% more than 2 assays over the year. The effect of age on the prescription patterns changes across each year of the 6-year study period are displayed in Fig. 1. It shows that the increase was observed for all age strata, but was particularly pronounced for the 50–80 ages groups with a peak in the 60–70 years old group. The main multivariable GEE poisson regression model showed a statistically significant association between each independent variable in the model and the incidence rate of vitamin D assays (Table 3). The most powerful variable explaining the increase was the calendar year. Beside, once adjusted for the calendar year, the change in vitamin D assay prescription rate was strongly associated with a low income (*CMU*) status with an IRR of 3.15 [CI95% 3.11–3.20], and moderately with the prescription of an OP (IRR = 1.54 [95% CI 1.51–1.56]), and the presence of severe comorbidities (versus absence) with an IRR of 1.25 [95% CI 1.24–1.27]. A significant non-linear association with age was confirmed. Conversely, changes in vitamin D assays prescription rate were almost not associated with the Charlson comorbidity index (IRR = 1.02 [95% CI 1.01–1.02], per unit increase) and very slightly with gender (women vs men IRR = 1.12 [95% CI 1.11–1.13]).

**Table 3:** Factors associated with increase of vitamin D assays prescriptions.

Patients characteristics	Vitamin D assays Incidence adjusted Rate Ratio (IRR*)	IC95%
Sex		
Male	Ref	
Female	1.12	1.11–1.13
Age		
<30	Ref	
30– < 40	1.11	1.09–1.14
40– < 50	1.22	1.20–1.25
50– < 60	1.34	1.31–1.36
60– < 70	1.45	1.42–1.48
70– < 80	1.51	1.48–1.54
80– < 90	1.36	1.33–1.39
> =90	1.14	1.10–1.19
*CMU* status		
Absence	Ref	
Presence	3.15	3.11–3.20
*ALD* status		
Absence	Ref	
Presence	1.25	1.24–1.27
Presence of OP		
Absence	Ref	
Presence	1.54	1.51–1.56
Charlson Comorbidity Index	1.02	1.01–1.02
Year		
2008	Ref	
2009	1.75	1.71–1.80
2010	3.27	3.19–3.36
2011	5.54	5.40–5.68
2012	6.96	6.78–7.14
2013	7.99	7.79–8.21

*Adjusted for all covariates displayed in the table.

Legend: This table presents the results of the Poisson regression aiming at describing factors associated with repetition of assay in a single patient. The analysis was performed on the study population, i.e patient having had at least one 25OHD assay during the study period.

## Discussion

The present study showed a strong and progressive increase in the frequency of testing for serum 25OHD and 1–25(OH)_2_D in the French population over the studied 6-year period, from 9,620 in 2008 to more than 81,641per year in 2013. The rise of testing was mostly due to an increased proportion of patients receiving at least one testing, to an increase in GP’s prescriptions and to a lesser extent in hospital specialists’ prescriptions. Vitamin D testing reimbursement incidence rate increased each year independently from age, comorbidities, and low income status. This shows that the trend of an increase observed in a single French hospital between 2007 and 2011 by Pilon *et al*. is observed at the national level^13^. Similar important changes have been noted in other countries, such as Canada^14^ and Australia, where Bilinski *et al*. reported a massive increase in the frequency of testing over an 11-year period, increasing from 40.6 tests/100,000 people in the year 2000 to 3472.2 tests/100,000 people in 2011^4^. As the increase tended to be greater in Australian states located at higher latitude and thus with higher deficiency prevalence, it has been suggested that this could be a consequence of preventative testing linked to increased awareness of the health benefits of achieving sufficient 25OHD status^4^. Another study conducted in Liverpool, UK, showed the same trends, with an 11 fold increase in request between 2007 and 2012^15^. They showed that the odds of detecting a 25OHD deficiency decreased progressively with the calendar year, with an odd of finding a deficient result 2.4 times higher in 2007 than in 2012. In another setting, Bilinski *et al*. showed that the magnitude of the rise in 25OHD testing did not translate into increased testing for physiological endpoints associated with 25OHD deficiency, such as osteoporosis^16^. Globally, whether this increase corresponds to an improvement of hypovitaminosis D management in UK and Australia is still largely unknown.

Beside the calendar year, increase in rate was independently associated with a low socio-demographic status in our study. Few data are available on factors associated with increase of 25-OHD assays prescriptions. In the study of Gowda *et al*. conducted in Australia in 1,217 patients to investigate testing patterns in primary care practice^17^ there was a moderate association between testing and being a migrant (IRR 1.19, [CI95 1.08–1.31], p < 0.05). That we noticed a strong association with a low income status may be consistent with Gowda results, however, as we did not have information on migrant status in our study we cannot go further in the interpretation of this fact.

New French clinical guidelines were published in 2011^18^. In every situation where the therapeutic goal requires an optimal serum 25OHD level for an appropriate medical care (e.g. osteoporosis), these guidelines recommend to measure the baseline level in order to define loading and maintenance dosages of vitamin D supplementation. In elderly patients aged 65 years or more, they recommend a systematic vitamin D supplementation with no biological testing, since the risk of low vitamin D levels is high while the risk of excessive dose is very low in this population. The impact on vitamin D assay prescription rates in elderly patients is unknown, but as the 60–70 year old patients are, according to our study, the age group where the increase in testing has been the most important, it is expected to be significant. The real economic impact of supplementation without 25OHD testing is also debated. Some authors have postulated that blanket supplementation would result in substantial reduction in global healthcare costs^19^, whereas others have shown that 25OHD deficiency, combined with lack of monitoring, predicted increased patients healthcare costs^20^.

The French National Authority in Health Evaluation (« *Haute Autorité de Santé* » (*HAS*)) recently published a report on the clinical usefulness of the 25OHD assay in the management of patients in numerous clinical situations^21^. Based on this report, the *HAS* considered 25OHD assays useless in many situations and recommends to limit reimbursements of the assays to only a few indications (i.e., rickets, osteomalacia, elderly patients with repeated falls, and surgical therapy of obesity in adults). These guidelines are considered as too restrictive by several experts who pointed out the risk of depriving patients of the testing they need^22, 23^. It will be interesting to follow the evolution of 25-OHD prescriptions over the years following the release of these guidelines as these are more prone to influence GP practitioners than expert opinions.

The main strength of this study is that it is based on recent data, extracted from a nationally representative sample of the exhaustive reimbursement database^8^, which validity and usefulness in pharmacoepidemiological studies have been previously studied and validated^24, 25^. This study is the description of the practice between 2008 and 2013 in prescription of vitamin D assays based on an exhaustive database involving all physicians in France. This precludes any selection bias of the physicians, unlike other descriptive studies based on samples of volunteer physicians or single centre study. Thus, we believe that this analysis provides a reasonably unbiased estimate of vitamin D assay use in the French primary care setting.

However, several limitations of the present study should be acknowledged. First, we were not able to obtain information on the precise reason for vitamin D assays prescriptions in individuals. Second, this study was based on claims obtained from a database generated primarily for administrative purposes, which is a type of database known to present some specific pitfalls to account for when analyzed in a research setting^26^. However these are usual drawbacks of medicoadministrative database which in turns are the only databases without selection bias.

## Conclusion

This study demonstrates a rapid and dramatic increase in 25OHD testing, mostly due to an increase of the proportion of patients tested versus an increase of repeated measures in individuals over time. The proportion of assays prescribed by GPs and hospital specialists increased over time. This increase was largely unexplained by the patients ’characteristics such as age, gender and comorbidities. It was more influenced by the low income status and probably by the practitioners’ characteristics but we did not have many information on these characteristics. This study provides additional evidence on the concern that 25OHD testing may be used inappropriately in practice. There is a need for studies to determine the drivers of this changing trend in 25OHD prescriptions and whether this increased testing translates into improved 25OHD status and subsequent health improvement in the French population.

### Availability of data and material

The data that support the findings are available from CNAMTS, but restrictions apply to the availability of these data, which were used under license for the current study, and so are not publicly available. Data are available upon request to and with permission of Institut des données de santé (IDS), contact: gipids@gip-ids.fr

### Ethics approval and consent to participate

Our national dataset, in accordance to the laws that regulate hospital database in France, namely articles L. 6113-7 et L. 6113-8 of the Public Health Code, ATIH (French agency of hospital information) and CNAMTS (French National Health Insurance Fund).

All legal conditions for epidemiological surveys were respected, and the French national commission governing the application of data privacy laws (the “Commission Nationale Informatique et Libertés”) issued approval for both projects. Since the study was strictly observational and used anonymous data, in accordance to the laws that regulate “non-interventional clinical research” in France, namely articles L.1121-1 and R.1121-2 of the Public Health Code, did not require the written informed consent from the participants or the authorization from any other ethics committee to conduct this survey.

## Electronic supplementary material

Supplementary information
