# Supplementary material for: Increase of vitamin D assays prescriptions and associated factors: a population-based cohort study
Source: Sci Rep. 2017 Sep 4;7:10361. doi: 10.1038/s41598-017-10263-8 (PMC5583252; doi:10.1038/s41598-017-10263-8)
Supplement: Supplementary file 1 — Supplementary information [file 41598_2017_10263_MOESM1_ESM.docx]

**Supplementary material: List of anti-osteoporotic drugs used in the study.**

| Spécialités | ATC | CIP7 | CIP13 |
| --- | --- | --- | --- |
| DIDRONEL 200MG CPR | M05BA01 | 3450985 | 3400934509858 |
| DIDRONEL 400MG CPR | M05BA01 | 3330620 | 3400933306205 |
| ETIDRONATE MYL 200MG CPR | M05BA01 | 3602082 | 3400936020825 |
| ETIDRONATE MYL 400MG CPR | M05BA01 | 3583025 | 3400935830258 |
| ETIDRONATE SDZ 400MG CPR | M05BA01 | 3641656 | 3400936525658 |
| CLASTOBAN 300MG/5ML SOL INJ AMP 5ML | M05BA02 | 3542322 | 3400935423221 |
| CLASTOBAN 800MG CPR | M05BA02 | 3620660 | 3400936206601 |
| LYTOS 520MG CPR | M05BA02 | 3404241 | 3400934042416 |
| AREDIA 15MG/5ML PDR ET SOL INJ | M05BA03 | 3494184 | 3400934941849 |
| AREDIA 60MG/10ML PDR ET SOL INJ | M05BA03 | 3494190 | 3400934941900 |
| AREDIA 90MG/10ML PDR ET SOL INJ | M05BA03 | 3494209 | 3400934942099 |
| OSTEPAM 15MG/1ML SOL INJ FL 1ML | M05BA03 | 3687960 | 3400936879607 |
| OSTEPAM 30MG/2ML SOL INJ FL 2ML | M05BA03 | 3687977 | 3400936879775 |
| OSTEPAM 60MG/4ML SOL INJ FL 4ML | M05BA03 | 3688008 | 3400936880085 |
| OSTEPAM 90MG/6ML SOL INJ FL 6ML | M05BA03 | 3688020 | 3400936880207 |
| PAMIDRONATE HPI 3MG/ML INJ FL 10ML | M05BA03 | 3816972 | 3400938169720 |
| PAMIDRONATE HPI 3MG/ML INJ FL 5ML | M05BA03 | 3816966 | 3400938169669 |
| PAMIDRONATE HPI 6MG/ML INJ FL 10ML | M05BA03 | 3816989 | 3400938169898 |
| PAMIDRONATE HPI 9MG/ML INJ FL 10ML | M05BA03 | 3816995 | 3400938169959 |
| PAMIDRONATE MYL 3MG/ML INJ FL 20ML | M05BA03 | 3650514 | 3400936505148 |
| PAMIDRONATE MYL 3MG/ML INJ FL 30ML | M05BA03 | 3650543 | 3400936505438 |
| PAMIDRONATE MYL 3MG/ML INJ FL 5ML | M05BA03 | 3650448 | 3400936504486 |
| PAMIDRONATE RTP 3MG/ML INJ FL 10ML | M05BA03 | 3712681 | 3400937126816 |
| PAMIDRONATE RTP 3MG/ML INJ FL 20ML | M05BA03 | 3712712 | 3400937127127 |
| PAMIDRONATE RTP 3MG/ML INJ FL 30ML | M05BA03 | 3712741 | 3400937127417 |
| PAMIDRONATE RTP 3MG/ML INJ FL 5ML | M05BA03 | 3712652 | 3400937126526 |
| ALENDRONATE TVC 10MG CPR | M05BA04 | 3665036 | 3400936650367 |
| ALENDRONIQUE ACIDE ACT 70MG CPR | M05BA04 | 4928667 | 3400949286676 |
| ALENDRONIQUE ACIDE ACT 70MG CPR | M05BA04 | 4928696 | 3400949286966 |
| ALENDRONIQUE ACIDE ALM 70MG CPR | M05BA04 | 3708863 | 3400937088633 |
| ALENDRONIQUE ACIDE ALM 70MG CPR | M05BA04 | 3708892 | 3400937088923 |
| ALENDRONIQUE ACIDE ALT 70MG CPR | M05BA04 | 3824983 | 3400938249835 |
| ALENDRONIQUE ACIDE ALT 70MG CPR | M05BA04 | 3825008 | 3400938250084 |
| ALENDRONIQUE ACIDE ARW 10MG CPR | M05BA04 | 3708716 | 3400937087162 |
| ALENDRONIQUE ACIDE ARW 70MG CPR | M05BA04 | 3708656 | 3400937086561 |
| ALENDRONIQUE ACIDE ARW 70MG CPR | M05BA04 | 3708679 | 3400937086790 |
| ALENDRONIQUE ACIDE BGA 70MG CPR | M05BA04 | 3708768 | 3400937087681 |
| ALENDRONIQUE ACIDE BGA 70MG CPR | M05BA04 | 3708780 | 3400937087803 |
| ALENDRONIQUE ACIDE EG 10MG CPR | M05BA04 | 3702211 | 3400937022118 |
| ALENDRONIQUE ACIDE EG 70MG CPR | M05BA04 | 3702168 | 3400937021685 |
| ALENDRONIQUE ACIDE EG 70MG CPR | M05BA04 | 3702180 | 3400937021807 |
| ALENDRONIQUE ACIDE EVO 70MG CPR | M05BA04 | 4192060 | 3400941920608 |
| ALENDRONIQUE ACIDE EVO 70MG CPR | M05BA04 | 4192083 | 3400941920837 |
| ALENDRONIQUE ACIDE ISD 70MG CPR | M05BA04 | 3914278 | 3400939142784 |
| ALENDRONIQUE ACIDE ISD 70MG CPR | M05BA04 | 3914290 | 3400939142906 |
| ALENDRONIQUE ACIDE MYL 70MG CPR | M05BA04 | 3743606 | 3400937436069 |
| ALENDRONIQUE ACIDE MYL 70MG CPR | M05BA04 | 3743629 | 3400937436298 |
| ALENDRONIQUE ACIDE PHR 70MG CPR | M05BA04 | 3914108 | 3400939141084 |
| ALENDRONIQUE ACIDE PHR 70MG CPR | M05BA04 | 3914120 | 3400939141206 |
| ALENDRONIQUE ACIDE QUA 70MG CPR | M05BA04 | 3743701 | 3400937437011 |
| ALENDRONIQUE ACIDE QUA 70MG CPR | M05BA04 | 3743724 | 3400937437240 |
| ALENDRONIQUE ACIDE RBX 70MG CPR | M05BA04 | 3837052 | 3400938370522 |
| ALENDRONIQUE ACIDE RBX 70MG CPR | M05BA04 | 3837075 | 3400938370751 |
| ALENDRONIQUE ACIDE RTP 70MG CPR | M05BA04 | 3708952 | 3400937089524 |
| ALENDRONIQUE ACIDE RTP 70MG CPR | M05BA04 | 3708975 | 3400937089753 |
| ALENDRONIQUE ACIDE SDZ 70MG CPR | M05BA04 | 3770141 | 3400937701419 |
| ALENDRONIQUE ACIDE SDZ 70MG CPR | M05BA04 | 3770164 | 3400937701648 |
| ALENDRONIQUE ACIDE TVC 70MG CPR | M05BA04 | 3749218 | 3400937492188 |
| ALENDRONIQUE ACIDE TVC 70MG CPR | M05BA04 | 3749230 | 3400937492300 |
| ALENDRONIQUE ACIDE TVS 70MG CPR | M05BA04 | 3914433 | 3400939144337 |
| ALENDRONIQUE ACIDE TVS 70MG CPR | M05BA04 | 3914462 | 3400939144627 |
| ALENDRONIQUE ACIDE ZYD 70MG CPR | M05BA04 | 3914551 | 3400939145518 |
| ALENDRONIQUE ACIDE ZYD 70MG CPR | M05BA04 | 3914574 | 3400939145747 |
| FOSAMAX 10MG CPR | M05BA04 | 3406731 | 3400934067310 |
| FOSAMAX 10MG CPR | M05BA04 | 5595914 | 3400955959144 |
| FOSAMAX 5MG CPR | M05BA04 | 3476275 |  |
| FOSAMAX 70MG CPR | M05BA04 | 3595637 | 3400935956378 |
| FOSAMAX 70MG CPR | M05BA04 | 3595666 | 3400935956668 |
| BONDRONAT 2MG/2ML SOL INJ FL 2ML | M05BA06 | 3656586 | 3400936565869 |
| BONDRONAT 6MG/6ML SOL INJ FL 6ML | M05BA06 | 3634857 | 3400936348578 |
| BONVIVA 150MG CPR | M05BA06 | 3716578 | 3400937165785 |
| BONVIVA 150MG CPR | M05BA06 | 3716584 | 3400937165846 |
| BONVIVA 3MG/3ML SOL INJ SER | M05BA06 | 3768718 | 3400937687188 |
| IBANDRONIQUE ACIDE TVC 150MG CPR | M05BA06 | 4985211 | 3400949852116 |
| IBANDRONIQUE ACIDE TVC 150MG CPR | M05BA06 | 4985228 | 3400949852284 |
| ACTONEL 5MG CPR | M05BA07 | 3543623 | 3400935436238 |
| ACTONEL 30MG CPR | M05BA07 | 3543669 | 3400935436696 |
| ACTONEL 35MG CPR | M05BA07 | 3615771 | 3400936157712 |
| ACTONEL 35MG CPR | M05BA07 | 3666685 | 3400936666856 |
| ACTONEL 75MG CPR | M05BA07 | 3845689 | 3400938456899 |
| ACTONEL 75MG CPR | M05BA07 | 3845703 | 3400938457032 |
| RISEDRONATE ACT 35MG CPR | M05BA07 | 4974101 | 3400949741014 |
| RISEDRONATE ACT 35MG CPR | M05BA07 | 4974124 | 3400949741243 |
| RISEDRONATE ALM 35MG CPR | M05BA07 | 4940941 | 3400949409419 |
| RISEDRONATE ALM 35MG CPR | M05BA07 | 4940958 | 3400949409587 |
| RISEDRONATE ALT 35MG CPR | M05BA07 | 2194020 | 3400921940206 |
| RISEDRONATE ALT 35MG CPR | M05BA07 | 2194043 | 3400921940435 |
| RISEDRONATE ARG 35MG CPR | M05BA07 | 4973461 | 3400949734610 |
| RISEDRONATE ARG 35MG CPR | M05BA07 | 4973478 | 3400949734788 |
| RISEDRONATE ARW 75MG CPR | M05BA07 | 4157307 | 3400941573071 |
| RISEDRONATE ARW 75MG CPR | M05BA07 | 4171945 | 3400941719455 |
| RISEDRONATE BGA 75MG CPR | M05BA07 | 4157282 | 3400941572821 |
| RISEDRONATE BGA 75MG CPR | M05BA07 | 4170383 | 3400941703836 |
| RISEDRONATE BGR 35MG CPR | M05BA07 | 4924540 | 3400949245406 |
| RISEDRONATE BGR 35MG CPR | M05BA07 | 4924563 | 3400949245635 |
| RISEDRONATE BLF 35MG CPR | M05BA07 | 4991849 | 3400949918492 |
| RISEDRONATE BLF 35MG CPR | M05BA07 | 4991855 | 3400949918553 |
| RISEDRONATE CRT 35MG CPR | M05BA07 | 4972504 | 3400949725045 |
| RISEDRONATE CRT 35MG CPR | M05BA07 | 4972527 | 3400949725274 |
| RISEDRONATE EG 35MG CPR | M05BA07 | 4972958 | 3400949729586 |
| RISEDRONATE EG 35MG CPR | M05BA07 | 4972964 | 3400949729647 |
| RISEDRONATE EG 5MG CPR | M05BA07 | 4972898 | 3400949728985 |
| RISEDRONATE EG 75MG CPR | M05BA07 | 4157313 | 3400941573132 |
| RISEDRONATE EG 75MG CPR | M05BA07 | 4171916 | 3400941719165 |
| RISEDRONATE EVO 35MG CPR | M05BA07 | 4978814 | 3400949788149 |
| RISEDRONATE EVO 35MG CPR | M05BA07 | 4978837 | 3400949788378 |
| RISEDRONATE ISD 35MG CPR | M05BA07 | 4973490 | 3400949734900 |
| RISEDRONATE ISD 35MG CPR | M05BA07 | 4973509 | 3400949735099 |
| RISEDRONATE MYL 35MG CPR | M05BA07 | 4974615 | 3400949746156 |
| RISEDRONATE MYL 35MG CPR | M05BA07 | 4974638 | 3400949746385 |
| RISEDRONATE PHR 35MG CPR | M05BA07 | 2193888 | 3400921938883 |
| RISEDRONATE PHR 35MG CPR | M05BA07 | 2193894 | 3400921938944 |
| RISEDRONATE PHR 75MG CPR | M05BA07 | 2221047 | 3400922210476 |
| RISEDRONATE PHR 75MG CPR | M05BA07 | 2221053 | 3400922210537 |
| RISEDRONATE QUA 35MG CPR | M05BA07 | 4974561 | 3400949745616 |
| RISEDRONATE QUA 35MG CPR | M05BA07 | 4974584 | 3400949745845 |
| RISEDRONATE RBX 35MG CPR | M05BA07 | 4911313 | 3400949113132 |
| RISEDRONATE RBX 35MG CPR | M05BA07 | 4927797 | 3400949277971 |
| RISEDRONATE RTP 35MG CPR | M05BA07 | 4969488 | 3400949694884 |
| RISEDRONATE RTP 35MG CPR | M05BA07 | 4969502 | 3400949695027 |
| RISEDRONATE SDZ 35MG CPR | M05BA07 | 4945708 | 3400949457083 |
| RISEDRONATE SDZ 35MG CPR | M05BA07 | 4945720 | 3400949457205 |
| RISEDRONATE SDZ 75MG CPR | M05BA07 | 2176370 | 3400921763706 |
| RISEDRONATE SDZ 75MG CPR | M05BA07 | 2176393 | 3400921763935 |
| RISEDRONATE TVC 35MG CPR | M05BA07 | 4961883 | 3400949618835 |
| RISEDRONATE TVC 35MG CPR | M05BA07 | 4961937 | 3400949619375 |
| RISEDRONATE TVC 75MG CPR | M05BA07 | 4169351 | 3400941693519 |
| RISEDRONATE TVC 75MG CPR | M05BA07 | 4169405 | 3400941694059 |
| RISEDRONATE WTR 35MG CPR | M05BA07 | 3534038 | 3400935340382 |
| RISEDRONATE WTR 35MG CPR | M05BA07 | 3534067 | 3400935340672 |
| RISEDRONATE ZEN 35MG CPR | M05BA07 | 3534038 | 3400935340382 |
| RISEDRONATE ZEN 35MG CPR | M05BA07 | 3534067 | 3400935340672 |
| RISEDRONATE ZYD 35MG CPR | M05BA07 | 4974897 | 3400949748976 |
| RISEDRONATE ZYD 35MG CPR | M05BA07 | 4974911 | 3400949749119 |
| ACLASTA 5MG/100ML SOL INJ FL | M05BA08 | 3658711 | 3400936587113 |
| ZOMETA 4MG/100ML SOL INJ FL 100ML | M05BA08 | 2175264 | 3400921752649 |
| ADROVANCE 70MG/140MCG CPR ; 70 MG/5600 UI | M05BB03 | 3820212 | 3400938202120 |
| ADROVANCE 70MG/140MCG CPR ; 70 MG/5600 UI | M05BB03 | 3820229 | 3400938202298 |
| ADROVANCE 70MG/70MCG CPR ; 70 MG/2800 UI | M05BB03 | 3780872 | 3400937808729 |
| ADROVANCE 70MG/70MCG CPR ; 70 MG/2800 UI | M05BB03 | 3780895 | 3400937808958 |
| FOSAVANCE 70MG/140MCG CPR ; 70 MG / 5600 UI | M05BB03 | 3820181 | 3400938201819 |
| FOSAVANCE 70MG/140MCG CPR ; 70 MG / 5600 UI | M05BB03 | 3820198 | 3400938201987 |
| FOSAVANCE 70MG/140MCG CPR (FR) ADP | M05BB03 | 4950193 | 3400949501939 |
| FOSAVANCE 70MG/140MCG CPR (UK) ADP | M05BB03 | 4950112 | 3400949501120 |
| FOSAVANCE 70MG/140MCG CPR (UK) ADP | M05BB03 | 4950158 | 3400949501588 |
| FOSAVANCE 70MG/70MCG CPR (FR) ADP | M05BB03 | 4950052 | 3400949500529 |
| FOSAVANCE 70MG/70MCG CPR (FR) ADP | M05BB03 | 4950187 | 3400949501878 |
| FOSAVANCE 70MG/70MCG CPR (UK) ADP | M05BB03 | 4950046 | 3400949500468 |
| FOSAVANCE 70MG/70MCG CPR (UK) ADP | M05BB03 | 4950141 | 3400949501410 |
| FOSAVANCE 70MG/70MCG CPR ; 70 MG/2800 UI | M05BB03 | 3692518 | 3400936925182 |
| FOSAVANCE 70MG/70MCG CPR ; 70 MG/2800 UI | M05BB03 | 3702234 | 3400937022347 |
| ACTONELCOMBI CPR + GLE SACHET ; 35 MG + 1000 MG/880 UI | M05BB04 | 3813057 | 3400938130577 |
| ACTONELCOMBI CPR + GLE SACHET ; 35 MG + 1000 MG/880 UI | M05BB04 | 3828455 | 3400938284553 |
| EVISTA 60MG CPR | G03XC01 | 3482726 | 3400934827266 |
| EVISTA 60MG CPR | G03XC01 | 3482732 | 3400934827327 |
| EVISTA 60MG CPR (UK) ADP | G03XC01 | 4950365 | 3400949503650 |
| EVISTA 60MG CPR (UK) ADP | G03XC01 | 4950371 | 3400949503711 |
| RALOPHARM 60MG CPR Gé | G03XC01 | 4199524 | 3400941995248 |
| RALOPHARM 60MG CPR Gé | G03XC01 | 4199547 | 3400941995477 |
| RALOXIFENE BGA 60MG CPR | G03XC01 | 4199470 | 3400941994708 |
| RALOXIFENE BGA 60MG CPR | G03XC01 | 4199493 | 3400941994937 |
| RALOXIFENE SDZ 60MG CPR | G03XC01 | 2211072 | 3400922110721 |
| RALOXIFENE SDZ 60MG CPR | G03XC01 | 2211095 | 3400922110950 |
| RALOXIFENE TVC 60MG CPR | G03XC01 | 4933504 | 3400949335046 |
| RALOXIFENE TVC 60MG CPR | G03XC01 | 4933510 | 3400949335107 |
| OPTRUMA 60MG CPR (UK) ADP | G03XC01 | 4950359 | 3400949503599 |
| OPTRUMA 60MG CPR (UK) ADP | G03XC01 | 4950388 | 3400949503889 |
| OPTRUMA 60MG CPR | G03XC01 | 3484843 | 3400934848438 |
| OPTRUMA 60MG CPR | G03XC01 | 3484866 | 3400934848667 |
| PROTELOS 2G GRANULES SACHET | M05BX03 | 3651703 | 3400936517035 |
| PROLIA 60MG/ML SOL INJ SER | M05BX04 | 4928578 | 3400949285785 |
| XGEVA 120MG/1,7ML SOL INJ FL | M05BX04 | 9384143 | 3400893841433 |
